# The wildland-urban interface raster dataset of Catalonia

**DOI:** 10.1016/j.dib.2017.12.066

**Published:** 2018-01-03

**Authors:** Fermín J. Alcasena, Cody R. Evers, Cristina Vega-Garcia

**Affiliations:** a Agriculture and Forest Engineering Department (EAGROF), University of Lleida, Alcalde Rovira Roure 191, 25198 Lleida, Catalonia, Spain; b Portland State University, Department of Environmental Science and Management, PO Box 751, Portland, OR 97207, USA; c Forest Sciences Centre of Catalonia, Carretera de Sant Llorenç de Morunys km 2, Solsona 25280, Catalonia, Spain

**Keywords:** Wildland-urban interface, Wildfire risk, Urban planning, Human communities, Catalonia

## Abstract

We provide the wildland urban interface (WUI) map of the autonomous community of Catalonia (Northeastern Spain). The map encompasses an area of some 3.21 million ha and is presented as a 150-m resolution raster dataset. Individual housing location, structure density and vegetation cover data were used to spatially assess in detail the interface, intermix and dispersed rural WUI communities with a geographical information system. Most WUI areas concentrate in the coastal belt where suburban sprawl has occurred nearby or within unmanaged forests. This geospatial information data provides an approximation of residential housing potential for loss given a wildfire, and represents a valuable contribution to assist landscape and urban planning in the region.

**Specifications Table**

**Table:** 

Subject area	Environmental sciences, forestry, urban planning
More specific subject area	Natural hazards
Type of data	Geospatial data
How data was acquired	*Does not apply*
Data format	Raster file (*.tif)
Experimental factors	*Does not apply*
Experimental features	We used a geographical information system (GIS) analysis to reclassify the residential housing at pixel level into different classes considering structure density and the surrounding vegetation.
Data source location	Autonomous community of Catalonia (Spain).
Data accessibility	The public repository of the University of Lleida: http://hdl.handle.net/10459.1/60480
Related research article	Martinuzzi, Sebastán; Stewart, Susan I.; Helmers, David P.; Mockrin, Miranda H.; Hammer, Roger B.; Radeloff, Volker C. 2015. The 2010 wildland-urban interface of the conterminous United States. Research Map NRS-8. Newtown Square, PA: U.S. Department of Agriculture, Forest Service, Northern Research Station. 124 p. [includes pull-out map]. https://doi.org/10.2737/NRS-RMAP-8.

**Value of the Data**

• Locations of valued assets within WUI can help prioritize risk mitigation activities at fine scales, including fuel treatments, ignition prevention programs, and evacuation or self-protection plans.
• These geospatial information data can be used to promote fire adapted communities when used in combination with fire modeling results and studies addressing social vulnerability.
• WUI maps can inform wildfire risk management in densely populated communities where large numbers of residential houses are exposed to recurrent wildfire risk.
• The WUI raster dataset can assist urban planning and policy making at a wide range of scales, from local to regional.

## 1 Data

**Fig. 1:**
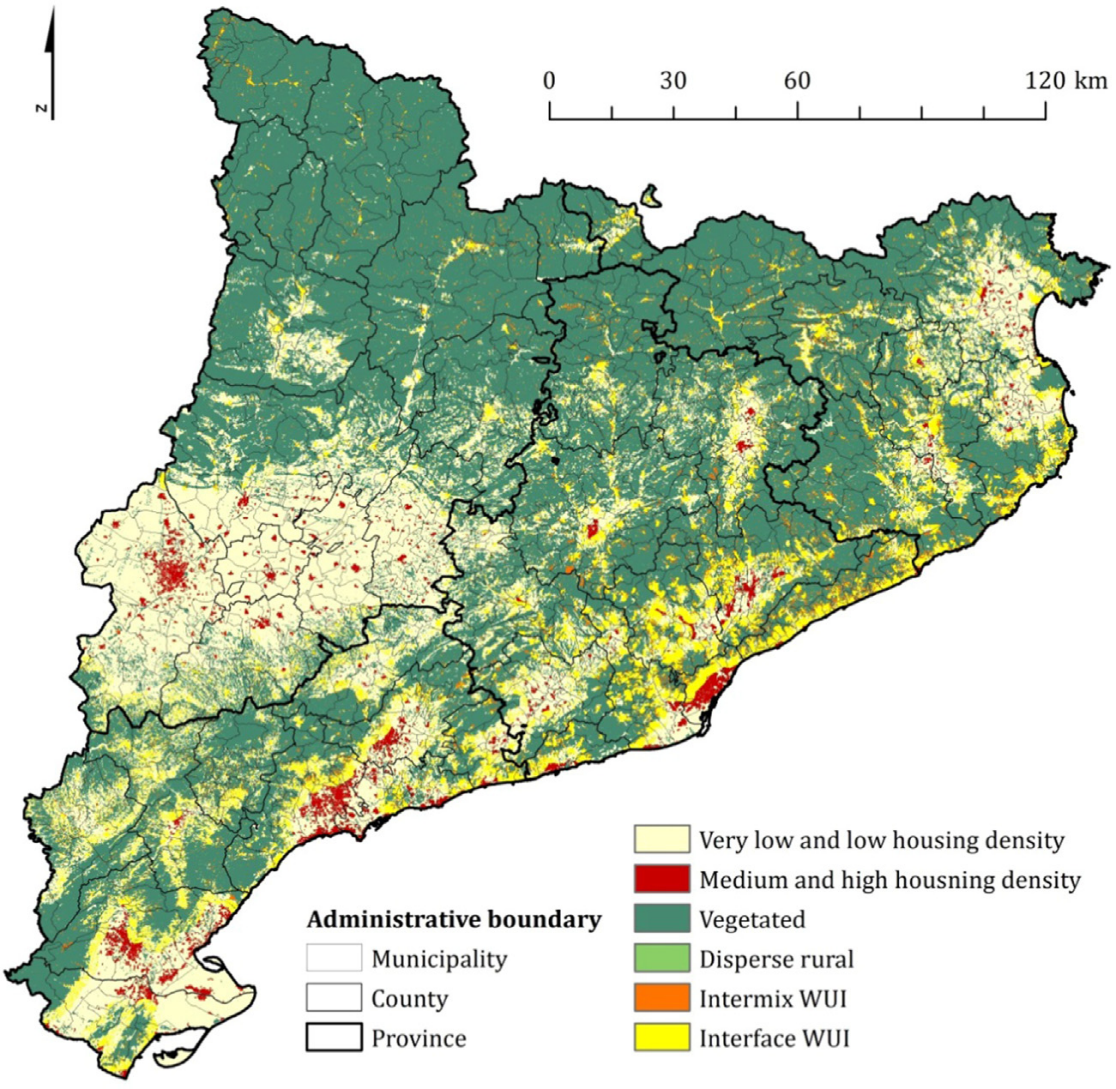
The wildland-urban interface (WUI) map of Catalonia (Northeastern Spain). The residential houses built in wilderness areas and inland traditional rural farming communities are typical examples of intermix WUI. Interface WUI communities are located primarily in highly populated metropolitan areas along the Mediterranean coast. See geospatial analysis section for further details about each WUI class.

The raster dataset of this article includes a detailed assessment (150-m resolution) of the wildland-urban interface for the 3.21 million ha autonomous community of Catalonia (Northeastern Spain) (Fig. 1). The WUI is the area where residential structures intermingle with hazardous vegetation and where most housing losses and human fatalities are concentrated in catastrophic wildfire events [1], [2], [3], [4]. This WUI raster map contains non-vegetated low housing density, non-vegetated high housing density, vegetated (no housing), dispersed rural housing, intermix housing and interface housing classes [5].

**Fig. 2:**
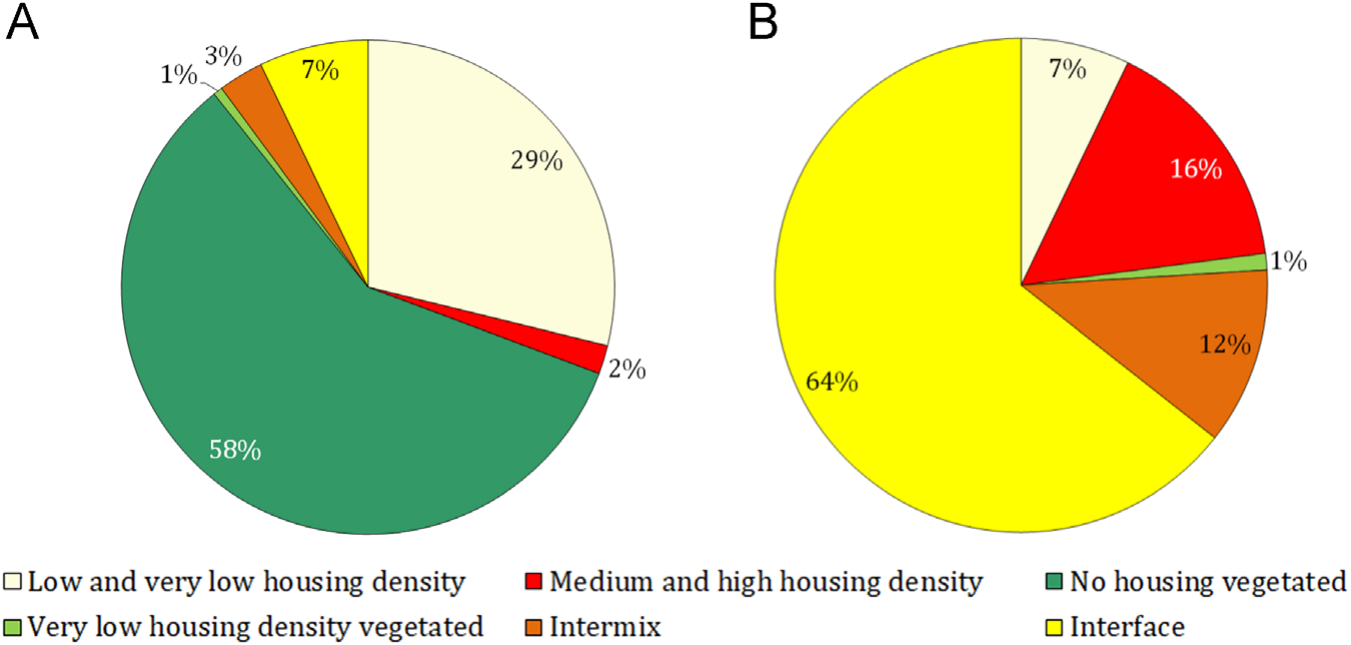
Proportions of WUI classes in Catalonia based on (A) total area and (B) structure count.

Dispersed rural, intermix and interface community classes respectively occupy 0.61% (19,559 ha), 2.96% (94,955 ha) and 7.16% (229,952 ha) (Fig. 2A) of Catalonia. Interface WUI occupies the widest areas in the coastal belt, and intermix WUI is more typical in central Catalonia and the Pre-Pyrenees region to the north. In the southwestern plain of Lleida, both interface and intermix WUI areas are limited due to large areas of irrigated agricultural lands. Here, only residential houses constructed on the transition edges between irrigation and dryland or forest patches are classified as WUI. Although the majority of residential house structures (>60%) are located in the interface WUI (*n*=517,571 structures), intermix (*n*= 93,113 structures) and disperse rural classes (*n*=8,693 structures) still account for a substantial number of structures at risk to wildfire (Fig. 2B).

## 2 Experimental design, materials, and methods

### 2.1 Residential housing and vegetation data

**Fig. 3:**
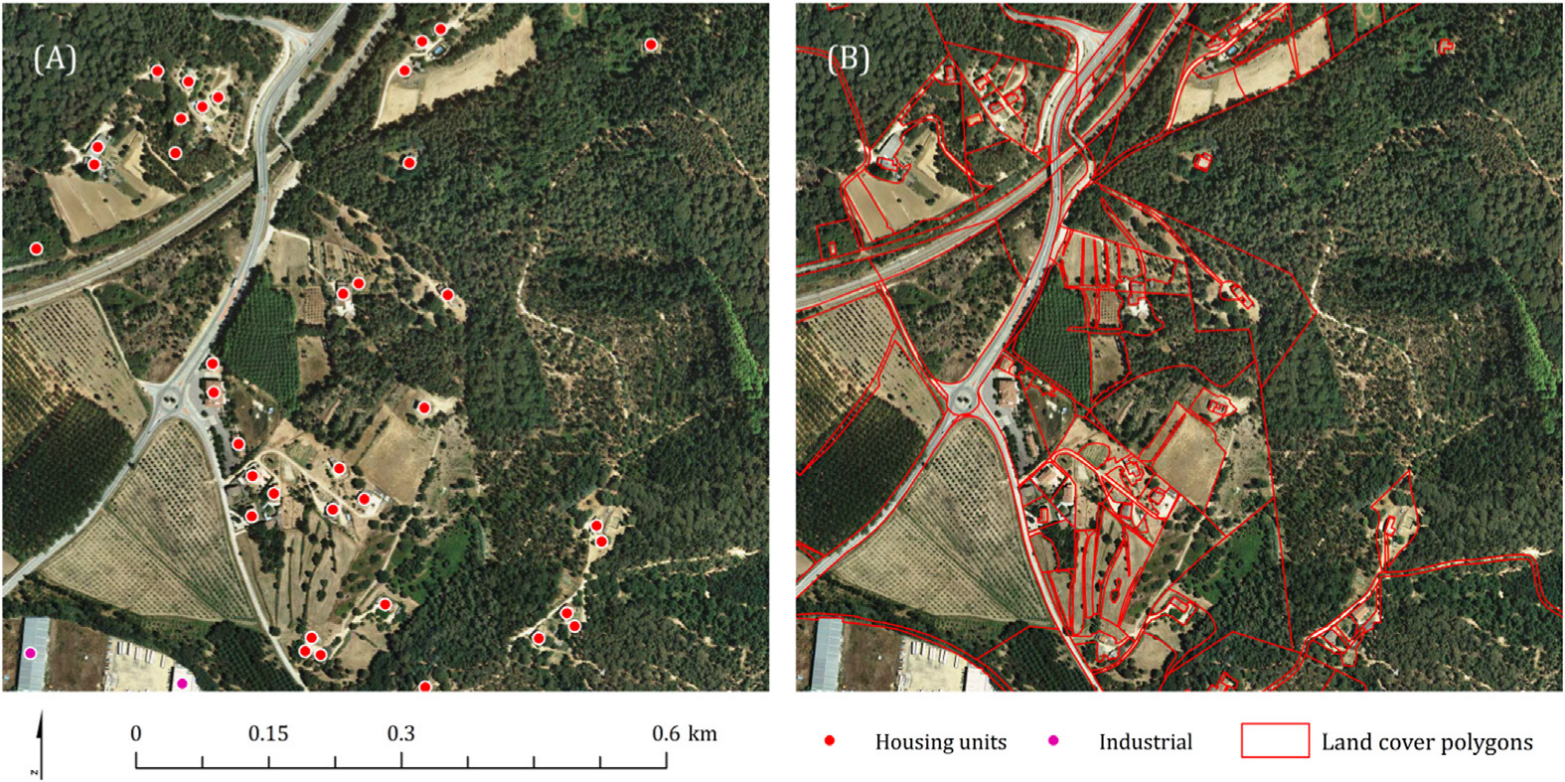
Closed-up view sample of the vegetation and structure location data used to generate de WUI map for Catalonia in the municipality of Massanes. (A) Individual housing structure centroids were used to calculate structure density [6]. (B) Land cover polygons were used to identify the forest land and tree covered-forested areas [7].

We mapped residential houses from the 1:25,000 scale Spanish national topographic platform (BTN25) map [6] (Fig. 3A). The BTN25 is the reference cartography used by municipalities and other official governmental agencies for multiple landscape and urban planning purposes and to accurately identify locations of individual structures. We did not distinguish between different type of residential houses (e.g., rural, housing block or chalet) and we excluded industrial structures, commercial buildings, and agricultural warehouses. We used the 2016 land parcel identification system (LPIS) vegetation map of Catalonia [7] to map areas where hazardous fuels can carry fire, torch stands, and threaten structures (Fig. 3B). We identified forest land polygons using the definition in Article 5 of the National Forest Law 43/2003, of 21 November, which includes natural pastures, shrubby pastures, open woodlands and tree covered-forested land.

### 2.2 Geospatial analysis

We used housing density, forest cover, and ember exposure grids in the WUI classification. Reference values for structure density and forest cover in the classification were obtained from similar studies that classified the WUI in other fire-prone areas [5], [8]. Risk exposure from ember showers was assumed to occur up to 2 km from forested lands based on the large-fire spotting distances observed in Catalonia [9]. All data were compiled at a 150 m resolution projected at ETRS89 UTM 31N coordinate system.

To construct the housing density layer, we first extracted individual residential house polygons from the BTN25 map (*n*=801,336) to generate a point file with structure location centroids. In the absence of census block information (a data source commonly used in similar US studies), we considered a 450 m regular grid (20.25 ha) as reference to calculate structure density at 150 m resolution. Pixels containing development were then reclassified as very low (<6.18 houses km^−2^), low (≥6.18–<49.42 houses km^−2^) and medium-high (≥49.42 houses km^−^^2^) density.

We generated the vegetation cover and ember exposure grids using the LPIS forest land polygons. To generate the vegetation cover grid we converted forest land polygons into a 150-m resolution raster grid and reclassified the pixels into vegetated (≥50% cover) and non-vegetated (<50% cover). Concurrently, to generate the ember exposure grid, we merged contiguous forested area polygons into larger blocks (the LPIS database subdivides polygons according to land ownership), and used resulting polygons >5 km^2^ to identify non-vegetated interface areas within a 2 km buffer that may be exposed to ember showers during catastrophic events.

Finally, we combined the three previous grids and assigned each 150-m raster grid cell as one of the 6 following classes:

– (1) Very low and low housing density: Forest land cover <50%, housing density ≤49.42 houses km^−^^2^, and >2 km from a forested land area ≥5 km^2^ in size.
– (2) Medium and high housing density: Forest land cover <50%, housing density ≥49.42 houses km^−2^, and >2 km from a forested land area ≥5 km^2^ in size.
– (3) Vegetated: Forest land cover ≥50% and no housing.
– (4) Dispersed rural: Housing density <6.18 houses km^−2^ and forest land cover ≥ 50%.
– (5) Intermix WUI: Housing density ≥6.18 houses km^−2^ and forest land cover ≥ 50%.
– (6) Interface WUI: Housing density ≥6.18 houses km^−2^, forest land cover <50% and houses located < 2 km from a forested area ≥ 5 km^2^ in size.
