# Supplementary material for: The wildland-urban interface raster dataset of Catalonia
Source: Data Brief. 2018 Jan 3;17:124–8. doi: 10.1016/j.dib.2017.12.066 (PMC5767903; doi:10.1016/j.dib.2017.12.066)
Supplement: Supplementary file 1 — Supplementary material [file mmc1.docx]

**CONFLICT OF INTEREST STATEMENT**

Manuscript tittle: **The Wildland-Urban Interface raster dataset of Catalonia**

Manuscript No.: DIB-D-17-01203R1

The authors whose names are listed immediately below certify that they have NO affiliations with or involvement in any organization or entity with any financial interest (such as honoraria; educational grants; participation in speakers’ bureaus; membership, employment, consultancies, stock ownership, or other equity interest; and expert testimony or patent-licensing arrangements), or non-financial interest (such as personal or professional relationships, affiliations, knowledge or beliefs) in the subject matter or materials discussed in this manuscript.

The corresponding author and responsible of the research:


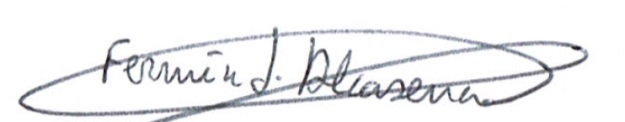


**Fermín J Alcasena Urdíroz** – Research Trainee Staff

Agriculture and Forest Engineering Department (EAGROF), University of Lleida,

Alcalde Rovira Roure 191, 25198 Lleida, Catalonia, Spain.
